# Eliminating deformation incompatibility in composites by gradient nanolayer architectures

**DOI:** 10.1038/s41598-018-34369-9

**Published:** 2018-11-01

**Authors:** Jianjun Li, Wenjun Lu, James Gibson, Siyuan Zhang, Tianyu Chen, Sandra Korte-Kerzel, Dierk Raabe

**Affiliations:** 1 State Key Laboratory of High Performance Complex Manufacturing, Central South University, Changsha, 410083 Hunan China; 2 College of Mechanical and Electrical Engineering, Central South University, Changsha, 410083 Hunan China; 3 Department of Microstructure Physics and Alloy Design, Max-Planck-Institut für Eisenforschung GmbH, Düsseldorf, 40237 Germany; 4 Institute of Physical Metallurgy and Metal Physics, RWTH Aachen University, Aachen, 52062 Germany; 5 Nanoanalytics and Interfaces, Max-Planck-Institut für Eisenforschung GmbH, Düsseldorf, 40237 Germany; 6 Department of Engineering Mechanics, Northwestern Polytechnical University, Xi’an, 710072 Shaanxi China

## Abstract

Composite materials usually possess a severe deformation incompatibility between the soft and hard phases. Here, we show how this incompatibility problem is overcome by a novel composite design. A gradient nanolayer-structured Cu-Zr material has been synthesized by magnetron sputtering and tested by micropillar compression. The interface spacing between the alternating Cu and Zr nanolayers increases gradually by one order of magnitude from 10 nm at the surface to 100 nm in the centre. The interface spacing gradient creates a mechanical gradient in the depth direction, which generates a deformation gradient during loading that accumulates a substantial amount of geometrically necessary dislocations. These dislocations render the component layers of originally high mechanical contrast compatible. As a result, we revealed a synergetic mechanical response in the material, which is characterized by fully compatible deformation between the constituent Cu and Zr nanolayers with different thicknesses, resulting in a maximum uniform layer strain of up to 60% in the composite. The deformed pillars have a smooth surface, validating the absence of deformation incompatibility between the layers. The joint deformation response is discussed in terms of a micromechanical finite element simulation.

## Introduction

Composite materials play a central role in the development of novel engineering solutions, particularly in the aerospace and automobile sectors^1,2^. A key goal in composite design is to utilize and combine the properties of the constituents, e.g., the deformability and lightness of the soft component and the high stiffness and strength of the hard component, to achieve the best advantages. For example, this combination is realized in metal matrix composites^3,4^ and carbon nanotube-reinforced composites^5–7^. However, composites are prone to severe deformation incompatibility between constituent phases that naturally possess a large mechanical mismatch. For example, the deformation incompatibility in metal matrix composites leads to pull-out of the carbon fibres^8,9^, the formation of cavities^10^, or premature fracture of the particulates or interfaces^11,12^. Another typical example is the shear instability observed in multilayered/phased nanostructures, such as shear cracks, shear bands or extrusion of soft materials^13–24^. These incompatibilities can drastically reduce the mechanical performance and service life of composite materials. Despite efforts to alleviate the incompatibility problem^25,26^, there is still no general strategy for eliminating deformation incompatibility in composite materials. We therefore designed a gradient nanolayer-structured composite material by the alternating stacking of soft Cu and hard Zr nanolayers with a gradient distribution in the layer thickness from the surface to the centre. The new design creates an interface spacing gradient structure. This design was inspired by the existing natural and artificial gradient structures, which show extraordinary deformability and compatibility^27–31^.

Natural gradient structures are ubiquitous^28^, such as the fibre density gradients in bamboo^32^, the alignment gradients in pangolin armour^33^ and the gradients in the chitin-calcite plywood structure of the exoskeleton of arthropods^34^. These gradients render biological materials resistant against harsh mechanical loads such as those imposed by weather or predators. Recently, manmade gradient structures have also been shown to provide improved strength-ductility properties^27,29–31,35–47^, lower friction coefficients^48^, better fatigue resistance^49–51^, and exceptional stretchability^52^. The underlying artificial gradient structures that were utilized in these studies mainly included grain size gradients spanning over four orders of magnitude^53^ or nanotwin gradients in twinning induced plasticity steel^54^. These gradient approaches have proven to be a successful strategy for enhancing the mechanical properties through defect gradients without requiring alloy modifications^55^, i.e., they were applied to pure metals or homogeneous solid solution alloys. Here, we extend this idea to a multiphase composite material in order to take the first step towards solving the general problem of the deformation incompatibility encountered in nanolayered composites by utilizing structural gradients. We used micropillar compression to test the deformation compatibility in the gradient architecture under nearly uniaxial stress conditions during deformation due to the fact that pillars have no confining material and, hence, reveal any deformation mismatch at the free surfaces upon mechanical loading.

Our specific approach for generally solving this deformation incompatibility problem lies in the design of composites that consist of a multiphase gradient nanostructure. The key idea behind this architecture is to give the layers with high mechanical contrast compatibility in their individual deformation, a process referred to as synergetic deformation^26^. We used geometrically necessary dislocations (GNDs) to achieve this goal. According to the classical work of Ashby^56^, GNDs accommodate a gradient of deformation in non-homogeneous materials and thus enable compatible deformation. The above conclusion has been validated by many scientists^57–61^. The aim of the nanolayer gradient design is to form a mechanical gradient in terms of strength and strain hardening, and in turn, non-uniform deformation in a gradient shape along the depth direction of the architecture. The ultimate goal is to generate sufficiently high populations of GNDs through gradient deformation to achieve strain compatibility between all the Cu and Zr layers with various thicknesses. Indeed, the new nanolayer gradient architecture enables large synergetic deformation (up to 60% uniform layer strain in the composite) between the layers with thicknesses varying from 10 nm to 100 nm. After loading, the pillars show a regular and smooth surface, indicating a considerably improved strain compatibility between the layers of soft Cu and hard Zr phases with various layer thicknesses. We analysed the results and the boundary conditions using a micromechanical finite element (FE) simulation, which models the individual layers and their co-deformation in terms of an elastic-plastic constitutive description (Supplementary Note 1).

## Results

### Microstructure of the gradient nanolayer architecture

**Figure 1:**
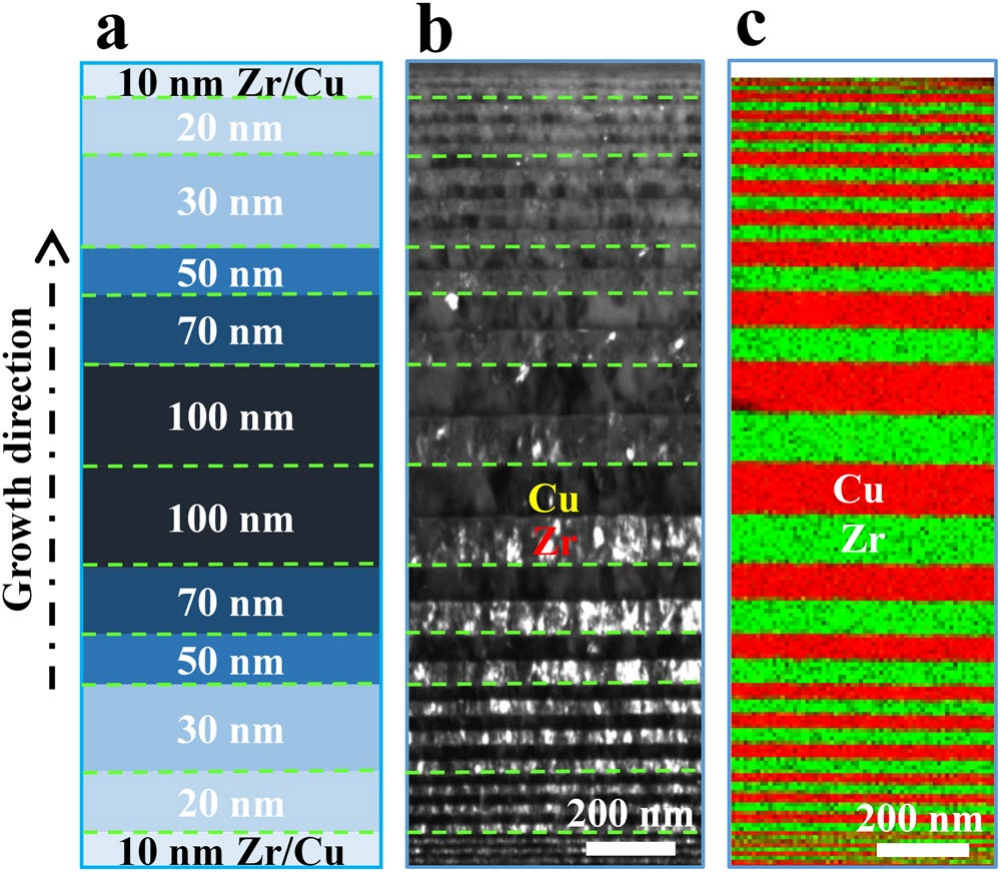
Microstructure of the gradient nanolayered (GNL) Cu/Zr material architecture that contains three 10 nm, 20 nm and 30 nm bilayers in both the top and bottom regions, i.e., sample GNL3. (**a**) Schematic sketch highlighting the spatial distribution of the bilayers consisting of one Zr layer and one Cu layer; (**b**) dark-field transmission electron microscopy (TEM) image; and (**c**) energy dispersive X-ray spectroscopy (EDS) map of Cu and Zr.

The gradient nanolayer (GNL) Cu/Zr composites were synthesized by magnetron sputtering (see Methods). The individual Cu or Zr layer thickness is designed to vary from 10 nm at the bottom to 100 nm in the centre and then from 100 nm back to 10 nm in the top half, thus producing an overall symmetric through-thickness layer distribution. Two gradient nanolayer samples, GNL1 and GNL3, were prepared, in which multiple Zr-Cu bilayers with constituent thicknesses of 10 nm, 20 nm and 30 nm were included in one half of the sample, and the numerals ‘1’ or ‘3’ indicate the number of the above bilayers of each thickness. The nominal total thicknesses of the two gradient samples are 1.12 μm and 1.6 μm, respectively. Figure 1 shows the microstructure of the as deposited gradient nanolayer sample containing three 10 nm, 20 nm and 30 nm Cu/Zr bilayers in both the top and bottom regions, i.e., GNL3. The dark-field transmission electron microscopy (TEM) image and the energy dispersive X-ray spectroscopy (EDS) map reveal the architectured gradient nanolayer structure and the compositionally sharp interfaces between Cu and Zr (Fig. 1b and c). The actual thickness of the 100 nm layers is 116.8 ± 2.4*nm* for Zr and 120.7 ± 1.4*nm* for Cu, while those of the 10 nm, 20 nm, 30 nm, 50 nm and 70 nm layers scale according to their nominal thickness. Moreover, Fig. 1b shows that there is a large amount of grain boundaries in the layers and the grain size scales with the layer thickness. As a result, the gradient distribution of the layer thickness also generates a corresponding grain size gradient along the sample depth. The X-ray diffraction (XRD) patterns are shown in Supplementary Fig. 1. There are five main peaks observed for Zr, i.e., $(10\bar{1}0)$, (0002), $(10\bar{1}1)$, $(11\bar{2}0)$, and $(10\bar{1}3)$, and two main peaks for Cu, i.e., (111) and (220).

### Deformation of the gradient nanolayer samples

**Figure 2:**
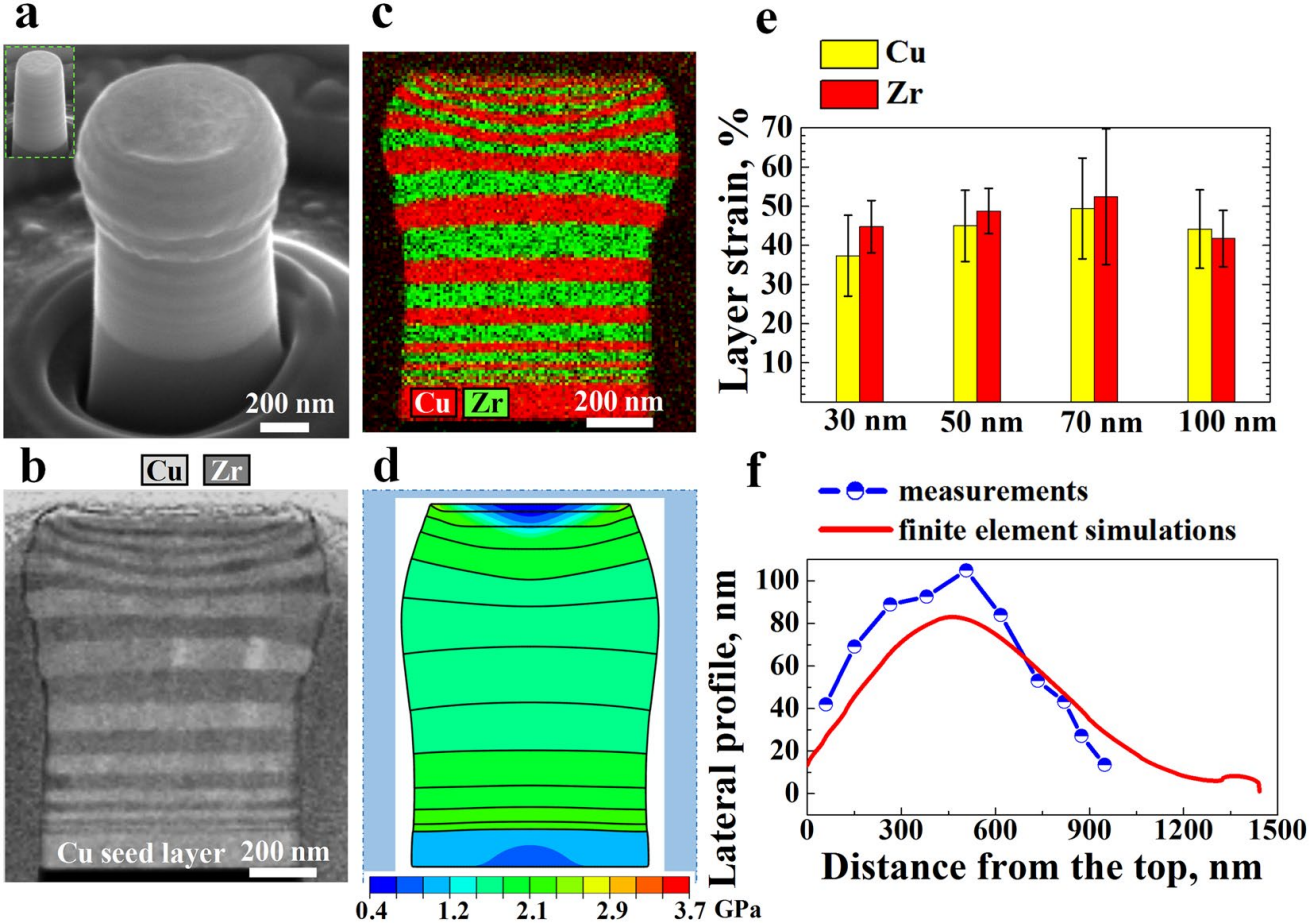
Deformation of the gradient nanolayered (GNL) sample that contains one 10 nm, 20 nm and 30 nm bilayer in both the top and bottom regions, i.e., sample GNL1, under a globally applied total strain of 22%. (**a**) Deformed morphology of the architectured composite with an undeformed pillar shown in the inset as a reference; (**b**) STEM image of the cross-section of (**a**); (**c**) corresponding EDS map of Cu and Zr; (**d**) deformed pillar with von Mises stress contour obtained from FE simulations, in which the interfaces between bilayers with different thicknesses are designated by lines; (**e**) average layer strains of Cu and Zr in the top half of the pillar; and (**f**) lateral profiles determined from measurements and FE simulations.

**Figure 3:**
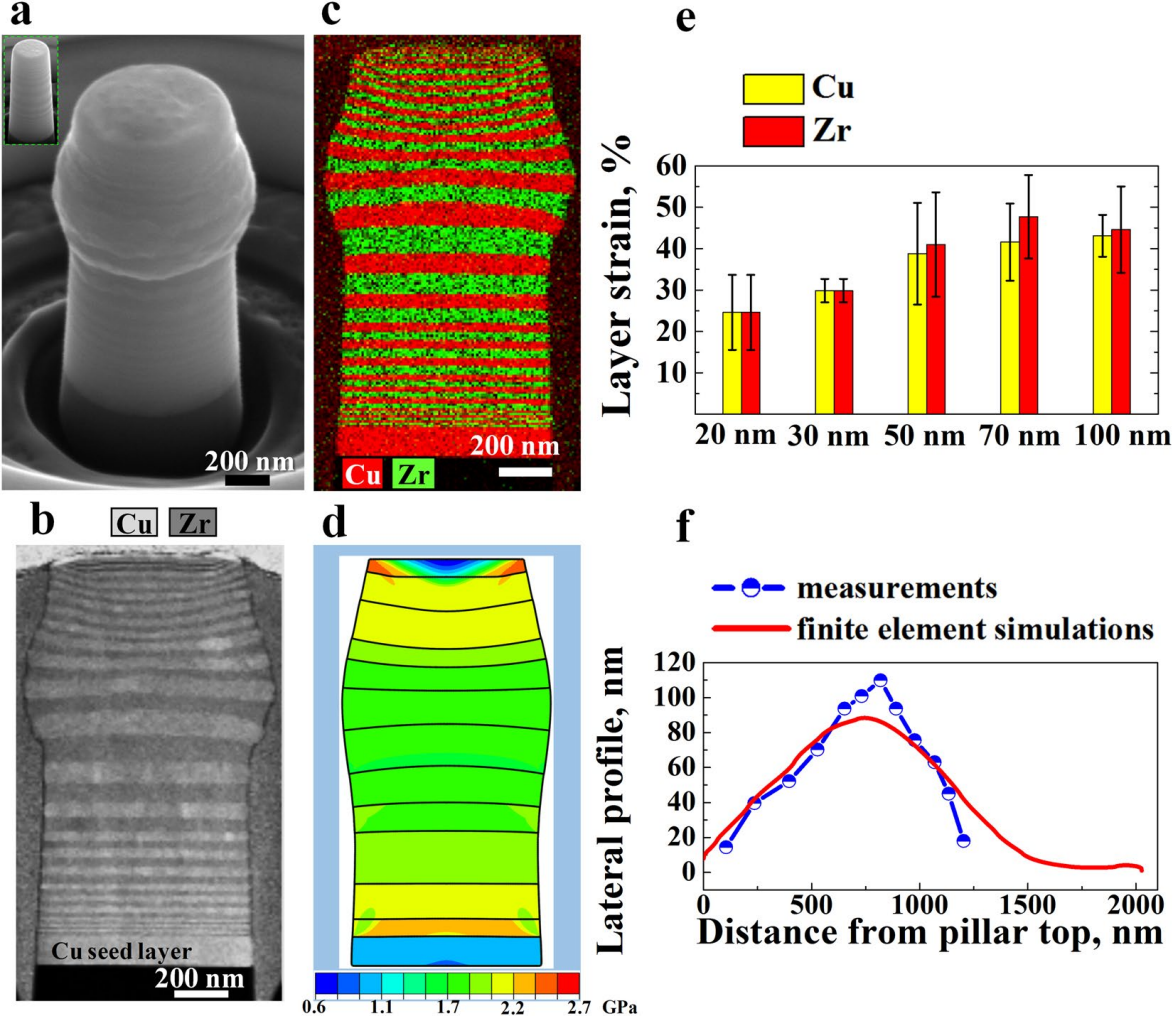
Deformation of the gradient nanolayered (GNL) sample containing three 10 nm, 20 nm and 30 nm bilayers in both the top and bottom regions, i.e., sample GNL3, under a globally applied strain of 20%. (**a**) Deformed morphology with an undeformed pillar (inset); (**b**) STEM image of the cross-section of (**a**); (**c**) corresponding EDS map of Cu and Zr; (d) deformed pillar with von Mises stress contour obtained from FE simulations, in which the interfaces between bilayers with different thickness are designated by lines; (**e**) average layer strain for Cu and Zr in the top half of the pillar; and (**f**) lateral profiles determined from measurements and FE simulations.

Figure 2 presents the deformation of sample GNL1, which contains only one 10 nm, 20 nm and 30 nm Cu/Zr bilayer in both the top and bottom zones of the sample. The sample was exposed to 22% total compressive strain. The Scanning TEM (STEM) image of the deformed pillar cross-section shows compatible deformation with even boundaries between all the smoothly co-deformed layers on both sides and no pronounced squeeze-out effects, here referred as synergetic deformation (Fig. 2b). The pronounced change in the gradient shape of the pillar is mainly due to the micromechanical effect induced by the gradient layered microstructure rather than the friction between the pillar top and the tool. The above friction induces only slight bulging in the top of the pillar that contains a homogeneous microstructure, whereas gradient-shape deformation occurs in the pillar with a gradient microstructure, as demonstrated by the finite element simulation (Supplementary Fig. 2). Moreover, the deformation in each layer is approximately uniform (Fig. 2b and c). The main effect of the friction is instead the introduction of small thickness variations in some layers of the top half of the pillar, which has also been observed in the FE simulations (Fig. 2d and 3d). This result means that the deformation is fully compatible between all the Cu and Zr layers, independent of the wide range of thicknesses spanning one order of magnitude and the associated nominal mechanical contrast. The previously reported strong extrusion and squeeze-out effects of Cu layers, as observed in earlier studies on homogeneous, viz., gradient-free nanolayer materials such as Cu/Zr^19,26,62^, Cu/Cr^63^ and Cu/amorphous CuZr^64–66^ systems, were not observed. The corresponding EDS maps of Cu and Zr also prove that no internal mixing, i.e., no severe cross-layer deformation, occurred (Fig. 2c). Moreover, the shear instability that propagates through several layers in the samples with thin layers (thickness less than 20 nm)^62^ has also been suppressed in the gradient composite due to the presence of the deformable, thick layers.

To better quantify and understand this homogeneous and synergetic co-deformation effect, we measured the individual thickness of each Cu and Zr layer from the top to the centre. Since the deformation concentrates in the top region because of the pillar taper and friction, only the top half of the pillar was measured. Additionally, we measured the thicknesses of only the 30 nm, 50 nm, 70 nm and 100 nm layers, where the measurements from the micrographs are sufficiently accurate. We made seven measurements at different locations in each layer to obtain an average layer thickness. The average layer strain was then calculated by dividing the change in the thickness of each layer before and after compression by the original thickness. Figure 2e presents the average strain of the individual Cu and Zr layers with different original thicknesses, i.e., 30 nm, 50 nm, 70 nm and 100 nm. First, the strain distributed gradually in the direction from the top of the pillar to the centre, which represents gradient deformation, as shown in Fig. 2b and c. Second, the average strains of the Cu and Zr layers with different original thicknesses are very close to each other. The strains of the Cu layers of different thicknesses are 37% ± 10%, 45% ± 9%, 49% ± 13% and 44% ± 10%, while those of Zr are 45% ± 7%, 49% ± 6%, 52% ± 17% and 42% ± 7%, which results in a maximum synergetic deformation of 51% ± 15% in the 70 nm layers. This value is calculated as the average of the strain of the Cu and Zr layers in the bilayer. The lateral profile shown in Fig. 2f also reveals a gradient in the deformation. Even when the globally applied strain increases to 31%, compatible deformation prevails, as shown by the smooth and continuous sample boundary after loading (Supplementary Fig. 3). The largest synergetic strain reaches 58% ± 10% in the 50 nm layers. Furthermore, the comparison of the simulated deformation morphologies (Fig. 2d and Supplementary Fig. 3d) and the micrographs (Fig. 2b,c and Supplementary Fig. 3b,c) shows that the deformed shapes and the lateral profiles are effectively predicted by the FE model for both globally applied strains, i.e., 22% and 31% (Fig. 2f and Supplementary Fig. 3f). Specifically, for the case of 22% applied strain, the highest lateral profile obtained from simulations is 83 nm, which occurs at 462 nm from the top, while the measurement gives the highest lateral profile of 105 nm, which occurs 507 nm from the top (Fig. 2f). The small quantitative discrepancy might result from the utilization of the approximate stress-strain curves for the Cu/Zr bilayers with different thicknesses as the input in the FE model.

Figure 3 shows the deformation of sample GNL3, which contains three 10 nm, 20 nm and 30 nm Cu/Zr bilayers in both the top and bottom areas. Similar to sample GNL1, GNL3 exhibits synergetic deformation with a smooth gradient boundary (Fig. 3b and c). We measured the average thicknesses of each individual Cu and Zr layer with thicknesses from 20 nm to 100 nm. The Cu and Zr layers in the refined layers (20 nm and 30 nm) were measured as one layer because it was difficult to distinguish the Cu and Zr layers in such thin bilayers after large deformation (Fig. 3b). Thus, we assume that the strains of Cu and Zr in the 20 nm and 30 nm bilayers are identical owing to their synergetic deformation. The maximum synergetic deformation reached 45%, occurring in the 70 nm layers (Fig. 3e). The gradient-shape deformation was also effectively predicted (Fig. 3d and f). However, when we increased the applied compression strain up to 31%, the extrusion or movement of Cu relative to Zr occurs in the 70 nm and 100 nm Cu-Zr bilayers in the lower half part of the pillar, as indicated by the arrows in Supplementary Fig. 4b and 4c. The deformation in other layers is still compatible. Unlike sample GNL1, the deformation of sample GNL3 shows incompatibility under a large applied strain, e.g., 31%. These findings show that an optimal gradient design exists, exemplified by sample GNL 1, which eliminates deformation incompatibility and ultimately achieves synergetic co-deformation, as validated by the smooth and continuous gradient boundaries observed in the compressed pillars (Fig. 2b and c and Supplementary Fig. 3b and c).

## Discussion

**Figure 4:**
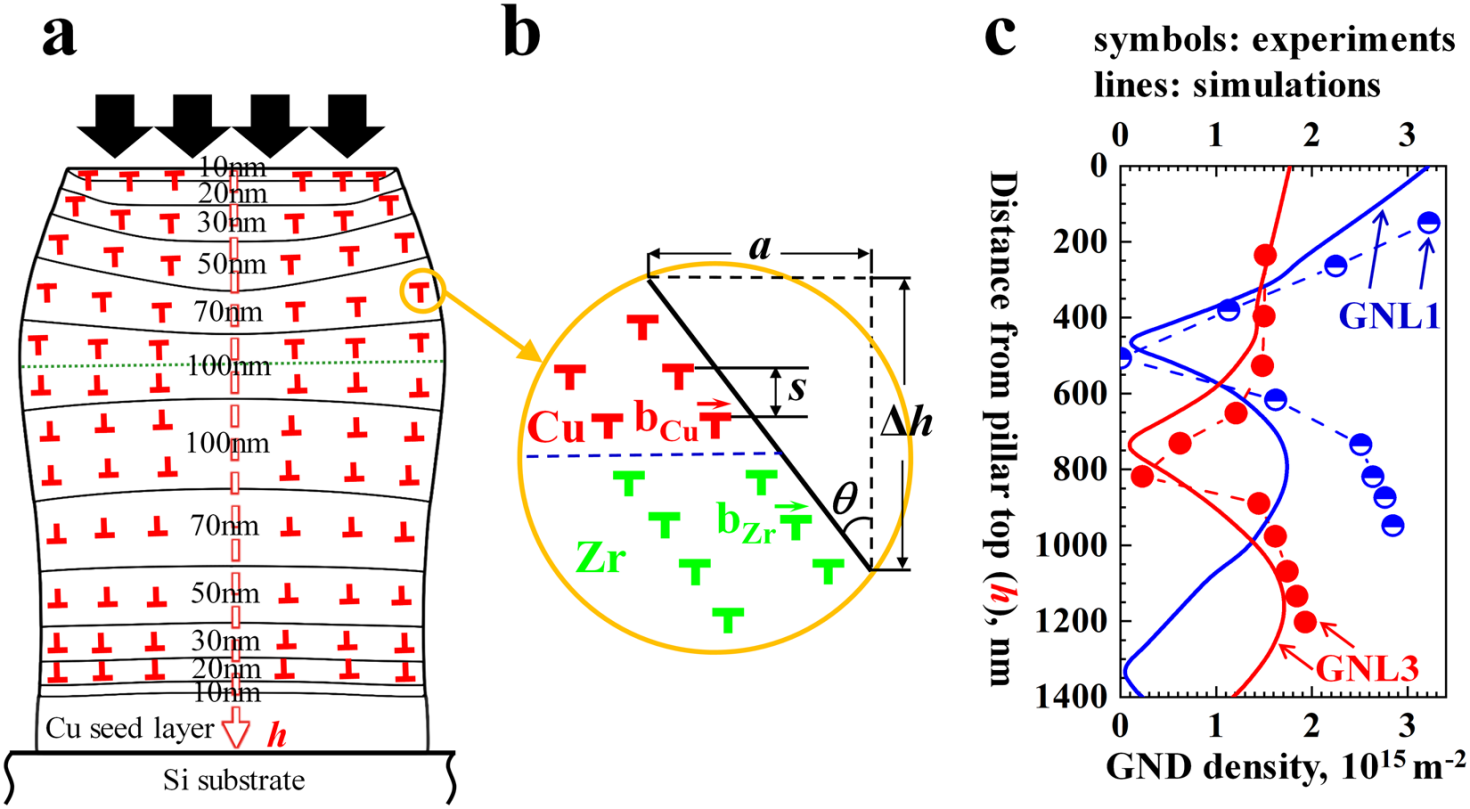
(**a**) Schematic of the accumulation of geometrically necessary dislocations (GNDs) along the sample depth *h*; (**b**) GNDs in Cu and Zr of an artificial layer; and (**c**) the calculated GND density along the thickness of samples GNL1 and GNL3 based on the lateral profiles obtained from experiments (symbols) and FE simulations (lines). The two samples are subjected to 22% and 20% global compression strains, respectively. The solid lines in (**a**) designate the interfaces of the Cu-Zr bi-layers with different constituent layer thicknesses (see the numerals).

The gradient distribution of the layer thickness is important for leading to this fully compatible deformation. The layer thickness gradient is accompanied by a grain size gradient. These two gradients generate a mechanical gradient in terms of the local yield strength and the strain hardening (see also Supplementary Table 1 and Supplementary Fig. 6). The mechanical gradient plays a key role in adjusting the otherwise high mechanical contrast between Cu and Zr. Moreover, as suggested by a plain dislocation-based model introduced recently to assess strain and stress compatibility in gradient nanograined materials^29,30^, the interplay between the gradients in local strain and the thickness-dependent mechanical size effects lead to the build-up of GNDs, enabling higher mechanical compatibility between the constituent layers. The accumulation of the GNDs along the sample thickness (*h*) accommodates the gradient deformation (Fig. 4a). All the GNDs are idealized as circular loops with Burgers vectors parallel to the interface plane. The change of the direction of the Burgers vector corresponds to the transition of the slope of the lateral profile from positive to negative. The whole structure is divided by many artificial layers with thickness Δ*h*, in each of which the GNDs are assumed to distribute uniformly (Fig. 4b), thus their density can be given as (Supplementary Note 2)

1 $${{\rho }}_{{\rm{G}}}=\frac{{\rm{2}}a}{bd{\rm{\Delta }}h}$$

where *a*, *b*, and *d* denote the difference in the lateral profile of neighbouring artificial layers, the magnitude of Burgers vector of GNDs in Cu or Zr layers, and the pillar diameter, respectively.

Figure 4c presents the calculated ${{\rho }}_{G}$ for samples GNL1 and GNL3 under ~20% globally applied strain based on the lateral profiles obtained from experiments and FE simulations. The simulation results show that with the increase of *h*, for both samples ${{\rho }}_{G}$ first decreases to zero, then it increases to a peak and finally decreases to zero again at the pillar bottom. The above trend agrees with the experimental derivation. The maximum value ${{\rho }}_{G}$ is obtained to be 3.21 × 10^15^m^−2^ in sample GNL1. This high dislocation density is generated to accommodate the compatible deformation among all the component layers with very different thicknesses and high mechanical contrast. Note that the overall GND density of sample GNL1 is larger than that of sample GNL3 though the two samples are subjected to approximately the same applied strain. The maximum ${{\rho }}_{G}$ in the former sample is nearly twice of that in the latter specimen, and the former possesses an average GND density of 1.26 × 10^15^ m^−2^, which is 24% higher than that in the latter, i.e., 1.01 × 10^15^ m^−2^. The higher GND density enables better deformation compatibility in sample GNL1 than sample GNL3 as observed in our experiments. As a result, the finding demonstrated a feasible approach to eliminate the deformation incompatibility in composites by generating sufficient GNDs through the gradient nanolayer design.

**Figure 5:**
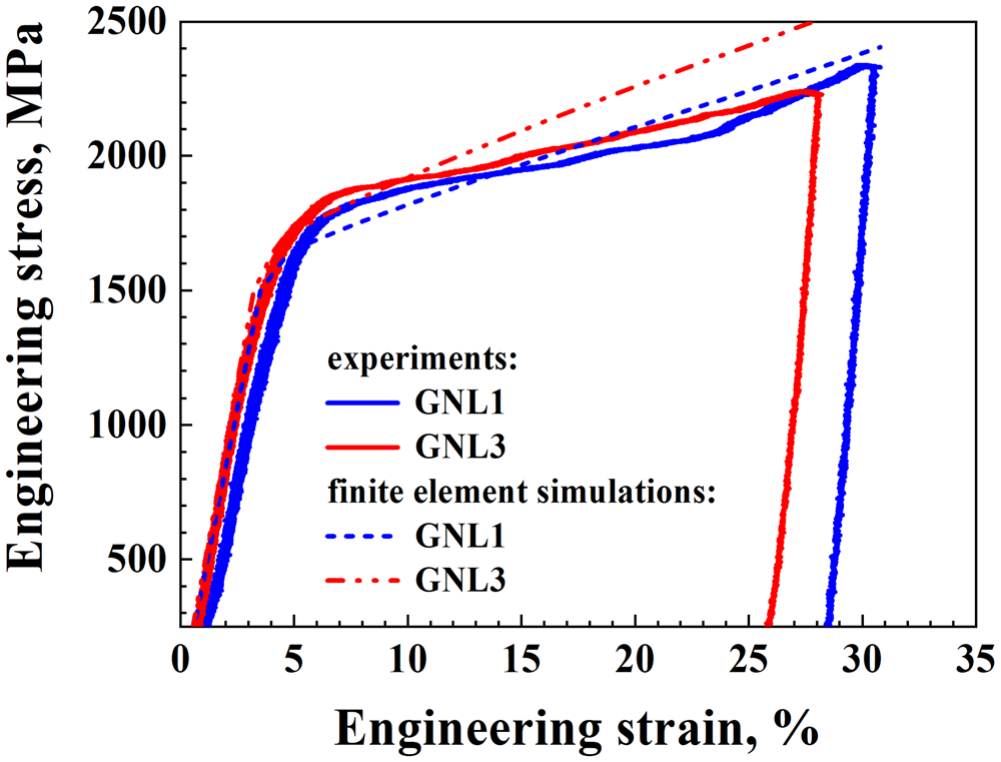
Typical engineering stress-engineering strain curves for gradient nanolayered (GNL) samples (GNL1 and GNL3). The curves obtained from finite element simulations are also included for comparison. The experimental drop in stress at strains >25% stems from unloading of the micropillar, not from any fundamental change in mechanism or boundary condition (e.g. necking).

Finally, the measured engineering stress-strain relations under compression are shown in Fig. 5. The flow stress of sample GNL3 is higher than that of sample GNL1 due to the larger fraction of thin layers (10 nm, 20 nm, and 30 nm) present in the former. The simulated curves are also included for comparison, revealing that the boundary condition treatment can approximately describe the overall mechanical response under compression. The experimental drop in stress at strains >25% stems from the sudden unloading of the micropillar, not from any fundamental change in the deformation mechanism or boundary condition (e.g. necking). The measured yield strength values of samples GNL1 and GNL3 are 1.94 GPa and 2.09 GPa, respectively. The slightly higher strain hardening (thus higher flow stress) observed in the simulations compared to the measurements might be due to the overestimated strain hardening behaviour of the component layers, as adopted in the FE model. These consistent phenomenological FE boundary condition simulations can be translated into underlying dislocation physics as follows: The used constitutive law (Eq. (S.2)) represents the geometrical confinement of a dislocation slip by the spaces between the heterointerfaces, represented by the factor *μb*/*h*. This part of the equation leads to an increase in the local resistance to plastic flow. In terms of the underlying dislocation physics, this means that this geometrical confinement necessarily leads to the gradual build-up of arrays of GNDs as a function of the thickness of the respective layers, i.e., thin layers are hardened much more than thick layers. In the current gradient architecture, this effect thus leads to a self-organized gradual adjustment and approximation of the flow stresses between the different layers, enabling the observed synergetic deformation.

In addition, the micropillar compression we used here approximates an isostress state. Hence, this method is an ideal way to test the deformation compatibility of the gradient structure. Preliminary nanoindentation tests up to a 500 nm depth showed that no shear band formation occurs in either of the gradient structures. As a result, it is conceivable that the synergetic deformation observed in the uniaxially compressed micropillars of the gradient architectures will also become a general phenomenon in other non-uniaxial tests with complex stress states, such as microcantilever bending and nanoindentation. Accordingly, the presented compatibility in the nanolayered composites may be modelled and hence exploited for tailoring the nanolayer design to achieve the maximum performance of these composites under the given loading conditions. Such an architecture may also be easily extended and realized on a large scale for engineering applications, e.g., by accumulative roll bonding^67–69^.

## Concluding Remarks

In summary, we designed a novel type of gradient nanolayer material in which co-deformation between the constituent nanolayers is enabled by their gradient thickness arrangement that spans over one order of magnitude from the surface to the centre. The key idea of designing a gradient nanoarchitecture is to avoid the localization that is otherwise characteristic of the originally high mechanical contrast between the layers of different thicknesses on the one hand and the Cu and Zr on the other. The aim is to accumulate substantial GNDs through the deformation gradient that is generated by the nanolayer thickness gradient. The GNDs ultimately give all the layers the best compatibility, which we called synergetic deformation. The maximum uniform synergetic deformation reaches 60% at a maximum yield stress of 1.9 GPa.

## Methods

### Materials preparation

The gradient nanolayered samples were synthesized by alternatively depositing Zr and Cu on a (100) silicon wafer through magnetron sputtering (Bestec PVD cluster). A 100 nm-thick Cu seed layer was deposited directly on the Si wafer before preparing the layered structure. The sputter targets are a Zr target of 99.9% purity and a Cu target of 99.99% purity. Both targets have a diameter of 76.2 mm and a thickness of 3 mm. Radio frequency sputtering is used to deposit Cu, whereas direct current sputtering is used to deposit Zr. The sputtering parameters were set as follows: the base pressure was less than 10^−7^ *mbar*; the work pressure was 3 × 10^−3^ *mbar*; the power was 100 W; and the rotation rate of the substrate was 20 rpm. The above set up results in a deposition rate of 0.1533 nm/s for Zr and 0.1467 nm/s for Cu.

### Micropillar preparation

The pillar was prepared by focus ion beam (FIB) milling (FEI nanolab 600 and 600i). The targeted diameter is 600 nm. In order to minimize the taper of the prepared pillar, we selected an appropriate set of annular patterns with decreasing outer (*D*) and inner (*d*) diameters with correspondingly reduced beam currents. The adopted diameters (*D* and *d*), cutting depth (*z*) and beam currents for all the patterns are listed in Supplementary Table 1. Approximately eight patterns were used. By using the listed parameters, 15–20 minutes are sufficient to prepare a pillar with taper less than 4°.

### Micropillar compression

Micropillar compression tests were carried out by using a 10-μm-diameter diamond flat punch (Nanomechanics InSEM-III) under a constant loading rate of 0.01 mN/s. The engineering stress-strain response was then calculated using the load-displacement records, taking the specimens’ cross-section at 20% of the pillar height from the top as a reference to translate the forces into stresses (Supplementary Note 3). The yield strength is adopted as the flow stress at 3% globally applied plastic strain.

### Preparation of pillar TEM lamella

The preparation of a TEM sample for the deformed pillar differs from that for a general TEM sample. We must ensure that the deformed pillar remains in the final TEM lamella. To achieve this goal, two strategies were made. The first one is to mark the position of the pillar by two rectangular patterns with size of approximately 5*μm* × 1*μm*. One pattern locates in the left side of the pillar and another in the right side. The used beam current was 48 pA. After marking, the pillar was coated successively by four annular patterns (Supplementary Table 2). Since the pillar was coated, the thickness of the protection Pt layer above the pillar top is unknown. To obtain an appropriately thick protection layer, we adopted the second strategy, that is, to measure the distance between the Si substrate and the protection layer surface after the first cut. The distance should be around 2 μm for pillars in sample GNL1 and 2.5 μm for those in sample GNL3 to contain both the deformed pillar and 1 μm-thick protective layer.

### Characterization of microstructure and deformation compatibility

The orientations and deformed morphologies were characterized by X-ray diffraction (XRD; GE Seifert 2-circle diffractometer) and scanning electron microscopy (SEM; FEI Helios Nanolab 600i). TEM and STEM were used to characterize the deformation compatibility of the pillars. TEM images, including diffraction contrast bright field and dark field images, were taken on a Philips CM20 microscope operated at 200 kV. STEM micrographs were obtained on a FEI Titan Themis microscope operated at 300 kV, using an aberration-corrected probe with ~0.1 nA current and a convergence semi-angle of 24 mrad, and the high-angle annular dark field (HAADF) detector with collection semi-angles of 73~352 mrad. We also collected the energy dispersive X-ray spectroscopy (EDS) spectra by a windowless, four quadrant silicon-drift-detectors covering a solid angle of 0.7 sr.

### Nanoindentation

A MicroMaterials Platform 3 nanoindenter with a diamond Berkovich indenter was used to measure the elastic modulus of the two gradient samples, i.e. GNL1 and GNL3. A total of nine indents were performed for each sample. The diamond area function of the tip was calibrated prior to indentation on a fused silica standard. A Poisson’s ratio of 0.33, i.e. the same as that of copper and close to that of Zr (0.34), was assumed for the conversion of reduced modulus to Young’s modulus.

## Electronic supplementary material

Supplementary material

## Data Availability

All data generated or analysed during this study are included in this published article (and its Supplementary Information file), or are available from the corresponding authors on reasonable request.
