# Supplementary material for: Eliminating deformation incompatibility in composites by gradient nanolayer architectures
Source: Sci Rep. 2018 Nov 1;8:16216. doi: 10.1038/s41598-018-34369-9 (PMC6212428; doi:10.1038/s41598-018-34369-9)
Supplement: Supplementary file 1 — Supplementary material [file 41598_2018_34369_MOESM1_ESM.docx]

**Supplementary Information**

**Eliminating deformation incompatibility in composites by gradient nanolayer architectures**

Jianjun Li^1,2,3,*^, Wenjun Lu^3^, James Gibson^4^, Siyuan Zhang^5^, Tianyu Chen^6^, Sandra Korte-Kerzel^4^ & Dierk Raabe^3^,[[1]](#footnote-1)

*^1^State Key Laboratory of High Performance Complex Manufacturing, Central South University, Changsha 410083, Hunan, China*

*^2^College of Mechanical and Electrical Engineering, Central South University, Changsha 410083, Hunan, China*

*^3^Department of Microstructure Physics and Alloy Design, Max-Planck-Institut für Eisenforschung GmbH, Düsseldorf 40237, Germany*

*^4^Institute of Physical Metallurgy and Metal Physics, RWTH Aachen University, Aachen 52062, Germany*

*^5^Nanoanalytics and Interfaces, Max-Planck-Institut für Eisenforschung GmbH, Düsseldorf 40237, Germany*

*^6^Department of Engineering Mechanics, Northwestern Polytechnical University, Xi’an 710072, Shaanxi, China*

**Supplementary Note 1. Finite element (FE) model of gradient nanolayered structures**

For a more consistent boundary condition analysis of the deformation in the gradient architectured nanolayer materials, we developed an axisymmetric finite element (FE) model (ABAQUS) for micropillar compression (Supplementary Fig. 5)[^1^](#_ENREF_1). We employed the *J*_2_ flow theory of plasticity for all the constituent layers, i.e., , where and $\sigma'$ are the plastic strain rate tensor and the deviatoric stress tensor, respectively, and is a factor that can be determined from uniaxial tensile tests, in which and $\sigma$ are respectively the equivalent von Mises plastic strain rate and stress. Due to the difficulty in measuring the mechanical response of each layer, power law relations (Eq. (S.1)) were used to model the equivalent Mises stress-strain response of each Cu/Zr bilayer with different individual layer thicknesses, i.e., 10 nm, 20 nm, 30 nm, 50 nm, 70 nm and 100 nm. Therefore, we have the following relation:

$\varepsilon^{p}=\frac{\sigma}{E}\left[\left(\frac{\sigma_{y}}{\sigma}\right)^{\left(N+1\right)}-1\right]$ (S.1)

The elastic modulus was obtained by nanoindentation (see Methods), i.e., *E*=132 GPa for sample GNL1 and *E*=125 GPa for sample GNL3, and $\sigma_{y}$ is the yield strength of each layer.

As the layer thickness decreases below 100 nm, the limited space can only facilitate the slip of single dislocations in the layer, referred to as confined layer slip. The refined confined layer slip model developed by Misra et al.[^2^](#_ENREF_2) can successfully describe the size-dependent yield strength of the layered structure with layer thicknesses ranging from several nanometres to 100 nm (e.g., Cu/Nb^[2](#_ENREF_2)^ and Cu/Zr^[3](#_ENREF_3)^ system). Thus, the refined confined layer slip model was used to calculate the yield strength $\sigma_{y}$ of the Cu/Zr bilayers according to the equation[^2^](#_ENREF_2)

$\sigma_{y}=M\frac{\mu b}{8\pi h'}\left(\frac{4-\nu}{1-\nu}\right)\ln\left(\frac{\alpha t'}{b}\right)-\frac{f}{h}+\frac{\mu b}{\left(1-\nu\right)\lambda}$ (S.2) where *M* is the Taylor factor; $t'=t/\sin\phi$ is the layer thickness parallel to the glide plane of the geometrically confined slip systems, where $\phi$ is the angle between the glide plane and the layer interface; *t* is the layer thickness; *b* and $\mu$ are respectively the magnitude of the Burgers vector and the shear modulus of Cu; $\lambda$ is the spacing between parallel arrays of gliding dislocation loops; and $\alpha$is the dislocation core cut-off parameter. The three terms in Eq. (S.2) represent the contributions of the confined layer slip, the interface stress and dislocation-dislocation interactions, respectively.

The values of the material parameters for calculating the yield strength $\sigma_{y}$ are listed in Supplementary Table 3. The resultant $\sigma_{y}$ values vary from 2.29 GPa to 1.1 GPa from the surface layer to the centre one (Supplementary Table 4). The adopted hardening exponent for all the component layers is sufficiently small, i.e., *N*=0.01, to model the low work hardening behaviour of the nanoscale layers. The calculated yield strengths of the 10 nm and 100 nm Cu/Zr layers agree with those measured by micropillar compression[^4^](#_ENREF_4) or our nanoindentation with a Tabor factor of 3. The yield strength of the 100 nm Cu seed layer is adopted as one third of the hardness of the 1.3-μm-thick Cu film. The input stress-plastic strain curves are presented in Supplementary Fig. 6.

Static mechanical analysis was conducted using the ABAQUS/Standard solver, in which 30701 and 44167 four-node axisymmetric elements (CAX4) were used for the cases GNL1 and GNL3, respectively. In the simulations the flat punch and the Si substrate were approximated as two analytical rigid bodies because the stiffness of the punch is almost one order of magnitude higher than that of the micropillar and the deformation is confined to the top half of the pillar due to the taper. The friction between the pillar top and the indenter was assumed to be infinite so that their relative movement could be eliminated according to experimental observations (Figs. 2 and 3). In order to avoid convergence problems, two rounded corners of 10 nm-radius were used in the upper and lower right side of the models (Supplementary Fig. 5). We modelled the Cu/Zr bilayer as a whole instead of modelling each constituent Cu or Zr layer since the bilayer strength can be approximately described by the refined confined layer slip model proposed by Misra^[2](#_ENREF_2)^. The constituent layer thicknesses of FE models are adopted by multiplying the nominal layer thickness by a factor of 1.2 according to the experimental measurements. A 120 nm-thick Cu seed layer is also included. The resultant pillar heights of GNL1 and GNL3 are 1464 nm and 2040 nm, respectively. Tapers of 2.7 and 2.2 degrees as measured were adopted for GNL1 and GNL3, respectively. In the simulation setup, all the constituent layers were fully bonded considering that the component layers showed compatible deformation in the experiments.

**Supplementary Note 2. Derivation of the density of geometrically necessary dislocations accumulated in the gradient nanolayered composites**

The geometrically necessary dislocations (GNDs) are assumed to be spaced equally along the sample depth and distributed uniformly in each artificial layer (Figs. 4a and 4b). The thickness of the artificial layer, i.e., $\Delta h$, is sufficiently small so that the layer has a straight boundary. By assuming that there are *n* GNDs in each row of the artificial layer, we have:

$\tan\theta=\frac{a}{\Delta h}=\frac{nb}{s}$ (S.3)

where $\theta$, *a*, *b*, *s* are the angle made by the accumulation of GNDs, the difference in lateral displacement between neighboring layers, the magnitude of Burgers vector of Cu or Zr, and the spacing between the individual slip steps, respectively (Fig. 4b). The expression of *s* can be obtained through Eq. (S.3), i.e.,

$s=\frac{nb}{a}\Delta h$ (S.4)

Assuming that the total length of the GND circular loops in each artificial layer is $\zeta$, the GNDs occupy an area of $\zeta s$, which can be also given based on a geometrical analysis, i.e.,

$\zeta s=\frac{\pi d\Delta h\left(n+1\right)}{2}$ (S.5)

in which *d* is the diameter of the micropillar. By substituting Eq. (S.4) into Eq. (S.5), we have

$\zeta=\frac{\pi da}{2b}\frac{\left(n+1\right)}{n}$ (S.6)

Generally, the value of *n* is very large, thus we have $\left(n+1\right)/n\approx1$ , leading to a simplified expression for $\zeta$, i.e.,

$\zeta=\frac{\pi da}{2b}$ (S.7)

Since the GNDs are assumed to distribute uniformly in each artificial layer, the volume occupied by the GNDs is

$V=\pi\left(\frac{d}{2}\right)^{2}\Delta h=\frac{\pi d^{2}\Delta h}{4}$ (S.8)

Therefore, the GNDs density in each artificial layer is

$\rho_{_{G}}=\frac{\zeta}{V}=\frac{2a}{bd\Delta h}$ (S.9)

In order to simplify the calculations without losing accuracy, the magnitude of the Burgers vector of Cu and Zr was adopted to be identical, i.e., *b*_Cu_=*b*_Zr_=0.256 nm. The pillar diameter, *d,* was adopted to be the same for all artificial layers due to the pillar taper being very small, and its value was set as the average of the top and bottom diameters of the pillar. The lateral profile data obtained from both experiments and FE simulations were smoothened before calculating the GND densities through Eq. (S.9).

**Supplementary Note 3. Calculation of engineering stress-strain curves**

The engineering stress is $\sigma=F/A_{0}$, in which *F* is the measured force and$A_{0}$ is the cross-sectional area of the prepared pillar. Considering that the deformation is confined to the pillar top due to the taper, *A*_0_ is set to be the cross-sectional area at 20% the pillar height from the top. The engineering strain can be calculated as $\varepsilon=L_{c}/H$, with *H* is the as prepared pillar height and $L_{c}$ the corrected displacement that can be written as[^5^](#_ENREF_5),[^6^](#_ENREF_6)

$L_{c}=L-\frac{1-v_{i}^{2}}{E_{i}}\left(\frac{F}{d_{i}}\right)-\frac{1-v_{s}^{2}}{E_{s}}\left(\frac{F}{d_{s}}\right)$ (S.10)

where *L* is the recorded displacement; $d_{i}$ and $d_{s}$ are the top and bottom diameters of the as prepared pillar, respectively; $E_{i}$ and $E_{s}$ are the Young’s modulus of the diamond indenter (1220 GPa^[7](#_ENREF_7)^) and that of the (100) Si substrate (130 GPa^[8](#_ENREF_8)^), respectively; $v_{i}\approx0.2$ and $v_{s}=0.278$[^9^](#_ENREF_9) are the corresponding Poisson’s ratios.

Supplementary Table 1. The annular patterns and beam currents used for preparing the 600 nm-diameter pillars.

| Order | *D* (μm) | *d* (μm) | *z* (μm) for reference | Beam current |
| --- | --- | --- | --- | --- |
| 1 | 30 | 15 | 0.1 | 9.3 nA |
| 2 | 15 | 7.5 | 0.1 | 9.3 nA |
| 3 | 8 | 2.5 | 0.1 | 2.5 nA |
| 4 | 6 | 2.5 | 0.1 | 2.5 nA |
| 5 | 4 | 1.2 | 0.1 | 80 pA |
| 6 | 3 | 1 | 0.1 | 80 pA |
| 7 | 1.5 | 1 | 0.1 | 80 pA |
| 8 | 1 | ～0.8 | 0.1 | 24 pA |

Supplementary Table 2. Annular patterns and beam currents used for preparing pillar TEM samples.

| Order | *D* (μm) | *d* (μm) | Beam current |
| --- | --- | --- | --- |
| 1 | 6 | 3 | 48 pA |
| 2 | 5 | 2.5 | 48 pA |
| 3 | 3 | 2 | 48 pA |
| 4 | 4 or 5 | 0 | 48 pA |

Supplementary Table 3. Yield strengths and exponents of Cu/Zr bilayers with different individual thicknesses and those of 100 nm Cu seed layer used in power law hardening constitutive relations.

|  | 10 nm | 20 nm | 30 nm | 50 nm | 70 nm | 100 nm | 100 nm Cu seed layer |
| --- | --- | --- | --- | --- | --- | --- | --- |
| $\sigma_{y}$ (GPa) | 2.29 | 2.11 | 1.96 | 1.79 | 1.69 | 1.61 | 1.1 |
| *N* | 0.01 | 0.01 | 0.01 | 0.01 | 0.01 | 0.01 | 0.01 |

Supplementary Table 4. Material parameters adopted in the finite element model for simulating the micropillar compression of gradient nanolayered structures.

| Parameter | Symbol | Magnitude |
| --- | --- | --- |
| Taylor factor | *M* | 3.06 |
| Shear modulus of Cu (GPa) | *μ* | 54.3 |
| Burgers vector magnitude of Cu (nm) | *b* | 0.256 |
| Poison’s ratio of Cu | *v* | 0.36 |
| Angle between the slip plane and interface () | *ϕ* | 70.5 |
| Core cutoff parameter | *α* | 0.09 |
| Spacing of a parallel array of glide dislocation loop (nm) | $\lambda$ | 16.75 |
| Interface stress parameter (*J*/*m*^2^) | *f* | 2 |


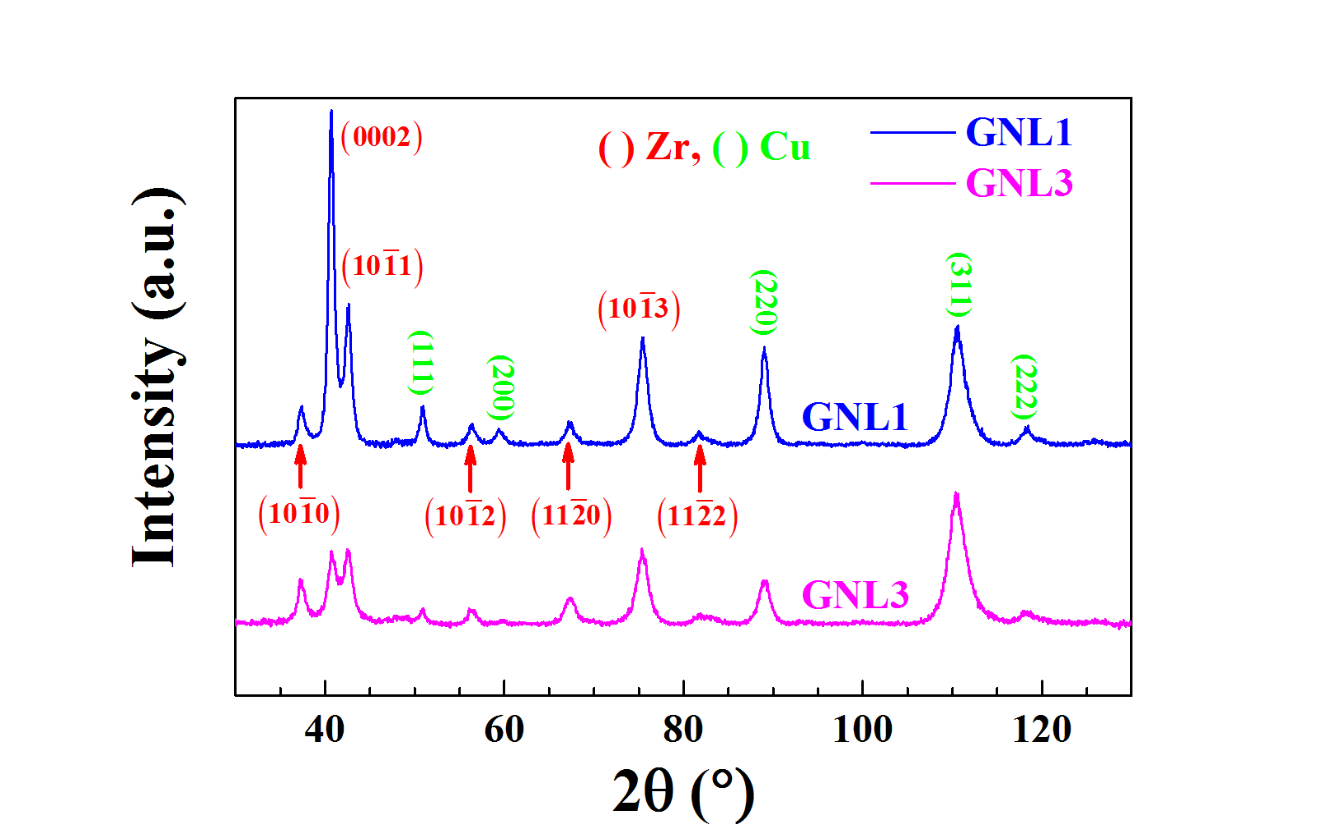


Supplementary Figure 1. XRD patterns for gradient nanolayered (GNL) samples (GNL1 and GNL3).


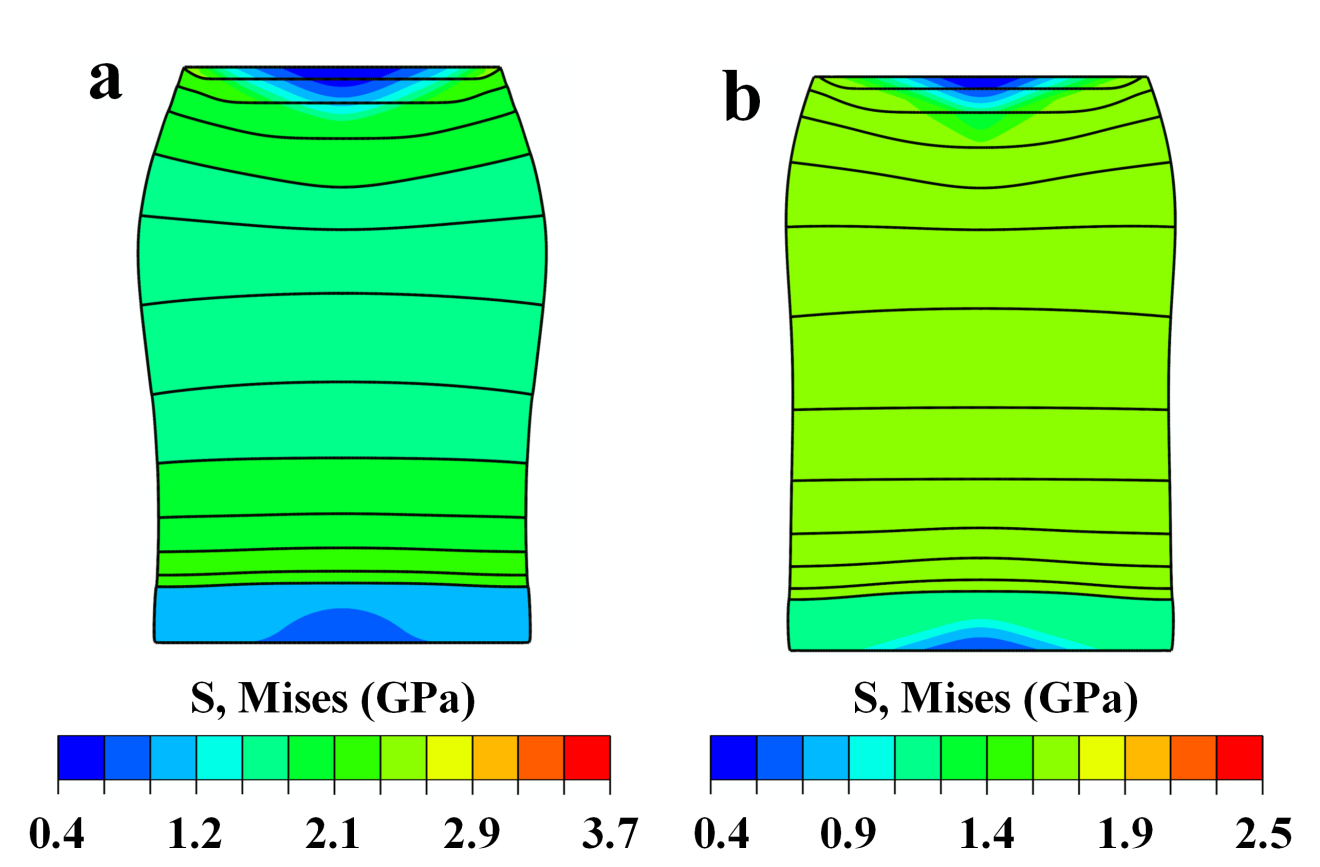


Supplementary Figure 2. Pillar deformation of gradient nanolayer (GNL) sample GNL1 (a) and homogeneous nanolayer structure using the 100 nm Cu/Zr material property (b) under 22 % globally applied strain.


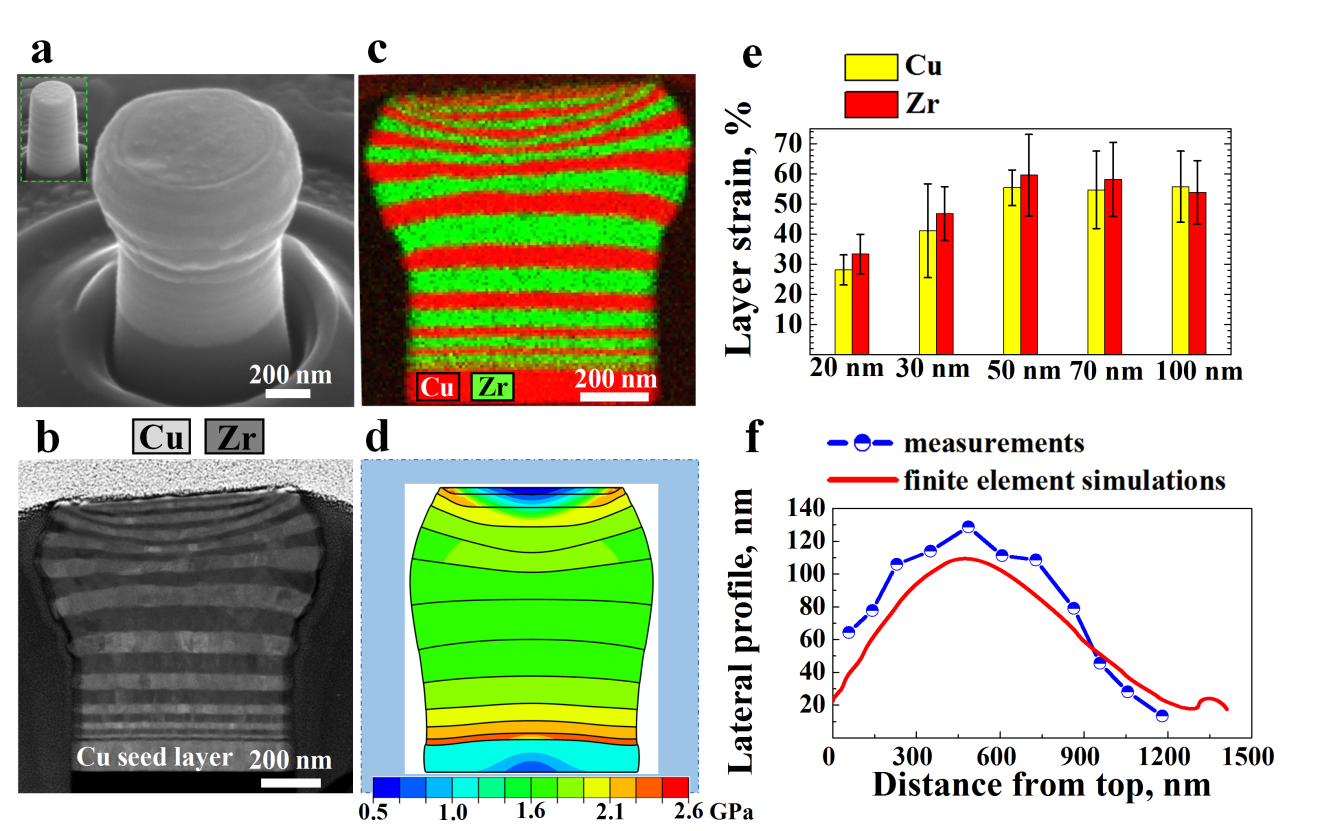


Supplementary Figure 3. Deformation of the gradient nanolayered (GNL) sample that contains one 10 nm, 20 nm and 30 nm bilayers in both the top and bottom regions, i.e., sample GNL1, under a globally applied strain of 31%. (a) Deformed morphology with an undeformed pillar (inset); (b) STEM image of the cross-section of (a); (c) corresponding EDS map of Cu and Zr; (d) deformed pillar with von Mises stress contour obtained from FE simulations, in which the interfaces between bilayers with different thickness are designated by lines; (e) average layer strain for Cu and Zr in the top half of the pillar; and (f) lateral profiles determined from measurements and FE simulations.


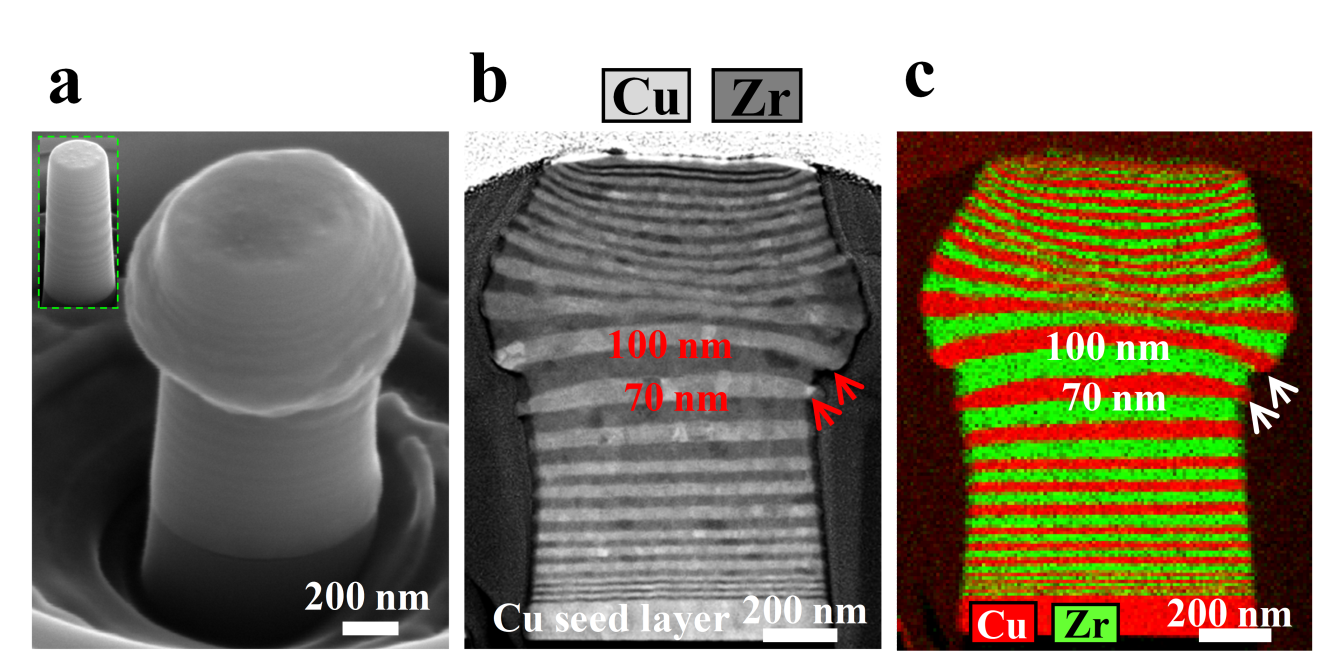


Supplementary Figure 4. Deformation of the gradient nanolayered (GNL) sample containing three 10 nm, 20 nm and 30 nm bilayers in both the top and bottom regions, i.e., GNL3, under a globally applied strain of 31%. (a) deformed morphology with an undeformed pillar (inset); (b) STEM image of the cross-section of (a); (c) corresponding EDS map of Cu and Zr.


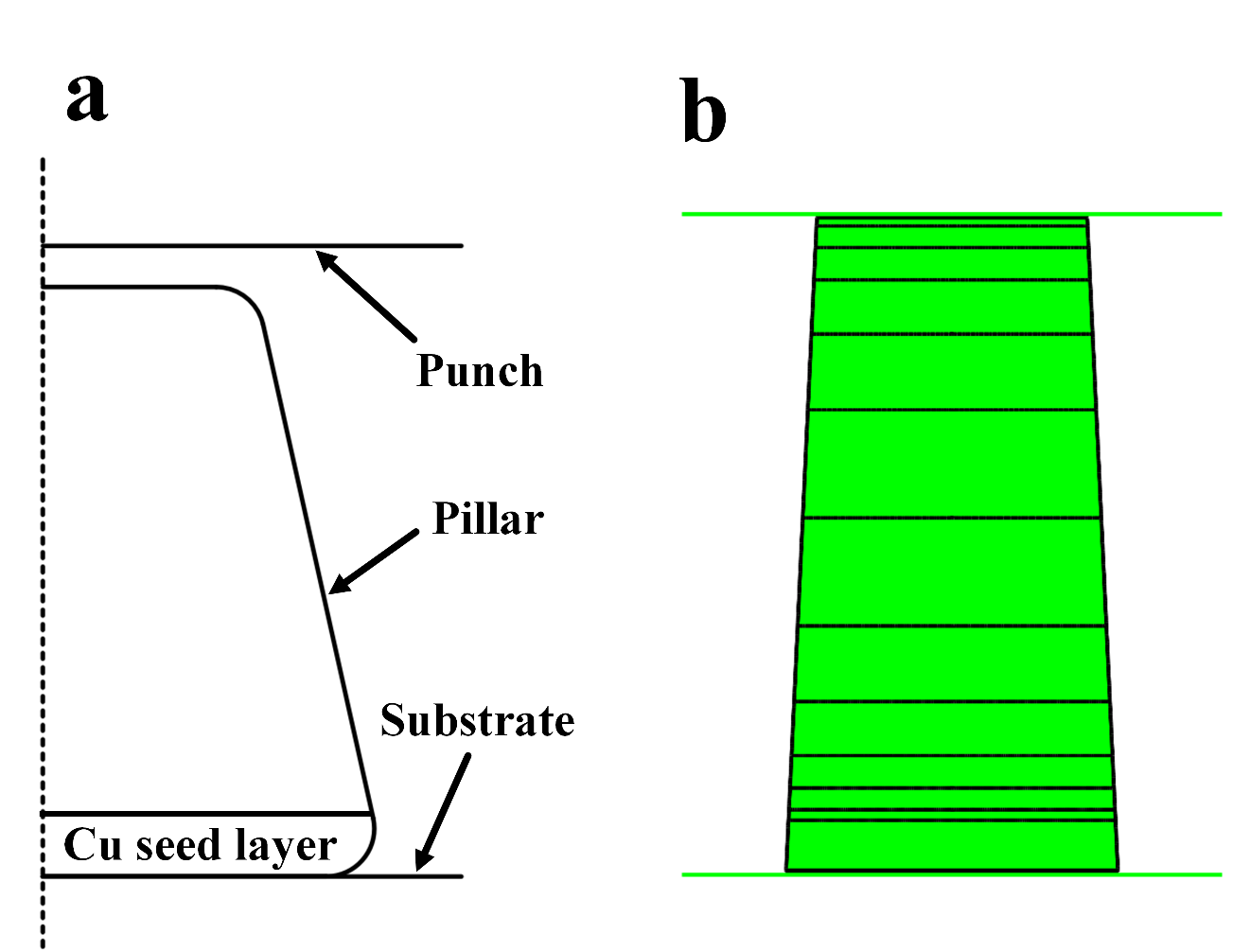


Supplementary Figure 5. Finite element (FE) model of gradient nanolayered structure: (a) Schematic; and (b) FE model for sample GNL1.

Supplementary Figure 6. Stress-strain data used in the finite element simulations for each Cu/Zr bilayer with different individual layer thickness.

**Supplementary References:**

1. Raabe, D., Ma, D. & Roters, F. Effects of initial orientation, sample geometry and friction on anisotropy and crystallographic orientation changes in single crystal microcompression deformation: A crystal plasticity finite element study. *Acta Mater.* **55**, 4567-4583 (2007).

2. Misra, A., Hirth, J. P. & Hoagland, R. G. Length-scale-dependent deformation mechanisms in incoherent metallic multilayered composites. *Acta Mater.* **53**, 4817-4824 (2005).

3. Zhang, J. *et al.* Length scale-dependent deformation behavior of nanolayered Cu/Zr micropillars. *Acta Mater.* **60**, 1610-1622 (2012).

4. Li, J., Lu, W., Zhang, S. & Raabe, D. Large strain synergetic material deformation enabled by hybrid nanolayer architectures. *Sci. Rep.* **7**, 11371 (2017).

5. Frick, C. P., Clark, B. G., Orso, S., Schneider, A. S. & Arzt, E. Size effect on strength and strain hardening of small-scale [1 1 1] nickel compression pillars. *Mater. Sci. Eng. A* **489**, 319-329 (2008).

6. Volkert, C. A. & Lilleodden, E. T. Size effects in the deformation of sub-micron Au columns. *Philos. Mag.* **86**, 5567-5579 (2006).

7. Spear, K. E. & Dismukes, J. P. *Synthetic diamond: emerging CVD science and technology*. Vol. 25 (John Wiley & Sons, 1994).

8. Hopcroft, M. A., Nix, W. D. & Kenny, T. W. What is the Young's Modulus of Silicon? *Journal of Microelectromechanical Systems* **19**, 229-238 (2010).

9. Gan, L., Ben-Nissan, B. & Ben-David, A. Modelling and finite element analysis of ultra-microhardness indentation of thin films. *Thin Solid Films* **290–291**, 362-366 (1996).

1. Corresponding authors.

   E-mail addresses: [mejjli@csu.edu.cn](mailto:mejjli@csu.edu.cn) (J. Li); [d.raabe@mpie.de](mailto:d.raabe@mpie.de) (D. Raabe) [↑](#footnote-ref-1)
